# Association between Periodontal Disease and Subsequent Sjögren’s Syndrome: A Nationwide Population-Based Cohort Study

**DOI:** 10.3390/ijerph16050771

**Published:** 2019-03-03

**Authors:** Chien-Yu Lin, Chien-Fu Tseng, Jui-Ming Liu, Heng-Chang Chuang, Wei-Te Lei, Lawrence Yu-Min Liu, Yu-Chin Yu, Ren-Jun Hsu

**Affiliations:** 1 Department of Pediatrics, Hsinchu MacKay Memorial Hospital, Hsinchu City 30071, Taiwan; mmhped.lin@gmail.com (C.-Y.L.); weite.lei@gmail.com (W.-T.L.); pockyfish@gmail.com (Y.-C.Y.); 2 Department of Dentistry and Oral Surgery, Taoyuan General Hospital, Ministry of Health and Welfare, Taoyuan 33004, Taiwan; guosentseng@gmail.com; 3 Division of Urology, Department of Surgery, Taoyuan General Hospital, Ministry of Health and Welfare, Taoyuan 33004, Taiwan; mento1218@gmail.com (J.-M.L.); chuang20110617@yahoo.com.tw (H.-C.C.); 4 Graduate Institute of Life Sciences, National Defense Medical Center, Taipei 11490, Taiwan; 5 Graduate Institute of Clinical Medical Sciences, College of Medicine, Chang Gung University, Taoyuan City 33302, Taiwan; 6 Department of Internal Medicine, Hsinchu MacKay Memorial Hospital, Hsinchu City 30071, Taiwan; drlawrenceliu@gmail.com; 7 Department of Medical Science & Institute of Bioinformatics and Structural Biology, National Tsing Hua University, Hsinchu City 30071, Taiwan; 8 Cancer Medicine Center of Buddhist Hualien Tzu Chi Hospital, Tzu Chi University, Hualien 97002, Taiwan; 9 Department of Pathology and Graduate Institute of Pathology and Parasitology, The Tri-Service General Hospital, National Defense Medical Center, Taipei 11490, Taiwan

**Keywords:** Sjögren’s syndrome, periodontal disease, national health insurance research database

## Abstract

Xerostomia (dry mouth) is the cardinal symptom of Sjögren’s syndrome (SS), which is an autoimmune disease involving the exocrine glands and other organs. Xerostomia may predispose patients to periodontal disease (PD) and an association between SS and PD has been reported. This association may be bidirectional; therefore, we conducted this study to investigate the risk of SS in patients with PD using data from the National Health Insurance Research Database of Taiwan. A total of 135,190 patients were enrolled in our analysis. In all, 27,041 patients with PD were matched by gender, age, insured region, urbanization and income, with cases and controls in a 1:4 ratio. Both groups were followed and the risks of SS were calculated by Cox proportional hazards regression. Finally, 3292 (2.4%) patients had newly diagnosed SS. Patients with PD had a significantly higher risk of subsequent SS (903 (3.3%) vs. 2389 (2.2%), adjusted hazard 1.47, 95% confidence interval: 1.36–1.59). In conclusion, patients with PD had an approximately 50% increased risk of subsequent SS. Physicians should be aware of the symptoms and signs of SS in patients with PD.

## 1. Introduction

Sjögren’s syndrome (SS) is an autoimmune disease characterized by exocrine gland dysfunction [1,2]. It is one of the most prevalent autoimmune diseases, with an estimated prevalence of 2–270 per 10,000 inhabitants [3,4,5]. The cardinal symptoms of SS are classical “sicca symptoms” of dry eyes (keratoconjunctivitis sicca) and dry mouth (xerostomia) but clinical manifestations may vary [2]. Usually, SS can be classified as either primary SS, which refers to involvement of solitary lacrimal and salivary glands or secondary SS, in which coexistence of other autoimmune diseases is observed, such as systemic lupus erythematosus and rheumatoid arthritis [1,2]. The heterogeneity of signs and symptoms often leads to a delay in diagnosis. Immune-mediated inflammatory processes are the main causes of SS and obvious lymphocytic infiltrates in salivary and lacrimal glands are observed [1,6,7,8]. Elevated autoantibodies, such as anti-Ro/SSA and anti-La/SSB, are common in patients with SS. Mucosal destruction and impairment of barrier function following immune-mediated inflammatory responses subsequently induce local inflammation and cause a vicious cycle. Extra glandular involvement of SS is common and increased risks of systemic diseases have been observed in patients with SS [9]. In patients with SS, increased risk of thyroid disease, cardiovascular diseases, gastrointestinal diseases, neuropsychiatric diseases and hematologic diseases were reported, and the systemic influences of SS raised our attention [10,11,12]. However, the underlying pathophysiology is complicated and not fully understood.

Periodontal disease (PD) is a common disease experienced by an estimated 20–50% of the global population [13,14]. Poor oral hygiene is believed to contribute to PD and patients with poor oral habits have a 2–5-fold increased risk of PD [13,15]. Patients with dry mouth, a smoking habit and diabetes also are at higher risk of PD. Poor oral hygiene results in colonization by oral microorganisms and causes local invasion, breakdown of barrier function and destruction of structures surrounding the teeth, including the gums, periodontal ligament or alveolar bone [13,16]. Furthermore, chronic oral inflammation may cause systemic inflammatory responses and alteration of the cytokine expression profile in serum or saliva have been reported, including interleukin (IL)-1, IL-6, IL-10, IL-17A, IL-17F, IL-22, IL-25, IL-33, tumor necrosis factor-alpha and interferon-gamma [17,18,19]. Long-term, systemic inflammation increases the risk of systemic diseases in patients with PD. Patients with PD were found to have a higher risk of cardiovascular diseases, maternal infection, preterm birth, low birth weight, preeclampsia and ulcerative colitis [14,20,21,22,23]. The important impact of PD on extra-oral systems is worth our attention.

Xerostomia is common in both diseases and immune alterations are evident in SS and PD. An association between SS and PD may exist. Several studies have investigated the risk of PD in patients with SS and they found that the mean plaque index, gingival index, bleeding on probing and mean gingival index were larger in patients with SS [24,25,26]. However, the evidence was not conclusive and the underlying pathophysiology was not clear [27,28]. The increased risk may be bidirectional and the risk of SS in patients with PD remains unclear. Therefore, we conducted this nationwide, retrospective cohort study to compare the risk of SS in patients with PD and a non-PD control group.

## 2. Materials and Methods

### 2.1. Study Design and DataBase

This study was approved by the Institutional Review Board of the Tri-Service General Hospital (approval number: TSGHIRB NO B-104-21). We extracted patient data from the National Health Insurance program of Taiwan, which is a nationwide medical insurance system with high coverage—99.5% of Taiwan’s 23 million residents—and highly representative of nationwide medical data [29,30]. Medical information regarding patient diagnosis and treatment-related information are included in the National Health Insurance Research Database (NHIRD). The International Classification of Diseases, 9th revision, Clinical Modification (ICD-9-CM) coding system was used in NHIRD and 1 million randomly selected individuals from the NHIRD in 2000 (LHID2000) were analyzed for our study. Patients with PD were selected as the study group and a matched group without PD was selected as the control group. Both cohorts were tracked to investigate the incidences of SS.

### 2.2. Selection Criteria and Study Flow Chart

**Figure 1:**
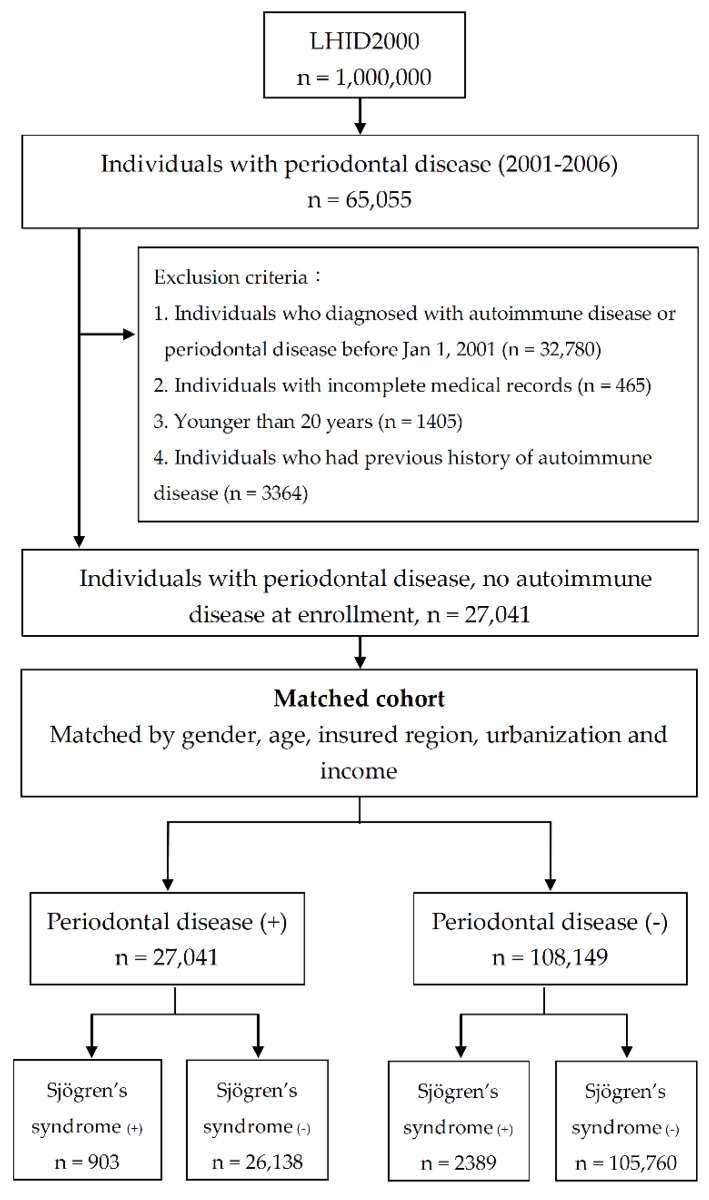
Flow chart illustrating the enrollment of study cohorts.

A total period of 13 years was investigated in our study and the flow chart of enrollment is shown in Figure 1. First, we identified patients with PD (ICD-9-CM: 523.3, 523.4 and 523.5) as the study group. The exclusion criteria included (1) history of PD before 2001, (2) incomplete medical records, (3) younger than 20 years and (4) previous history of autoimmune disease. The diagnosis was made by licensed dentists and mainly based on symptoms and local findings. The index date referred to the date of diagnosis of PD. Furthermore, a matched cohort was identified as the control group, with cases and controls in a 1:4 ratio. For each patient in the PD group, four matched controls were enrolled of the same gender, age, insured region, urbanization and income. Finally, we tracked both groups to identify patients newly diagnosed with SS (ICD-9-CM: 710.2) and compare the risk of SS in these two groups. SS was diagnosed mainly by rheumatologists according to clinical manifestations and laboratory tests. SS is a catastrophic illness in Taiwan and application for certification requires review by a second specialist. The index date referred to the date each individual was enrolled in the study. The censoring date referred to 7 years after the index date, death, the date of diagnosis of SS or lost-to-follow-up.

### 2.3. Study Outcomes and Covariates

Medical and demographic information for both cohorts was extracted from NHIRD and analyzed, including age, monthly income, geographic area of residence, urbanization level of residence and comorbidities. The primary outcome was newly diagnosed SS, a catastrophic illness in Taiwan. Age was divided into six groups based on 10-year intervals: 20 to 29, 30 to 39, 40 to 49, 50 to 59, 60 to 69 and ≥70 years. The monthly income of the study population was recorded in New Taiwan Dollars and categorized into four income levels. The geographic regions in Taiwan were divided into four areas: the northern region, central region, southern region and other region (eastern and outlying islands). The urbanized level of residence in Taiwan was classified into four categories. Finally, comorbid diseases included diabetes mellitus (DM, ICD-9-CM: 250), hypertension (ICD-9-CM: 401–405), hyperlipidemia (ICD-9-CM: 272), coronary artery disease (ICD-9-CM: 410–414), stroke (ICD-9-CM: 430–438), alcoholism (ICD-9-CM: 291, 303, 305.00–305.03, 571.1, 571.2, 571.3, 790.3, A215 and V11.3), obesity (ICD-9-CM: 278) and tobacco use disorder (smoking, ICD-9-CM: 305.1, 491.0, 491.2, 492.8, 496, 523.6, 649.0, 989.84 and V15.82). The incidences of newly diagnosed SS and the abovementioned covariates and comorbidities in both cohorts were investigated and analyzed.

### 2.4. Statistical Analysis

We used a Student’s *t*-test and a chi-square test to analyze and compare the categorical demographic characteristics and comorbidities of the two cohorts. Cox proportional hazards regressions were performed to evaluate the relationship between PD and subsequent SS. Moreover, the hazard ratio (HR) was calculated with the 95% confidence interval (CI) to compare the risk of PD. Further adjustment for potential confounders (age, gender, income, geographic area of residence, level of urbanization of residence and comorbidities) was performed in all models and the adjusted HR (aHR) was calculated. A two-sided *p* value < 0.05 was considered to indicate a statistically significant result. Statistical analyses were performed using SPSS software version 19.0 (SPSS Inc., Chicago, IL, USA) and data were managed with Microsoft^®^ SQL Server^®^ 2008 software (Microsoft Unternehmen, Redmond, DC, USA).

## 3. Results

As shown in Figure 1, 27,041 patients with PD were identified as the study cohort. We matched each individual in the study cohort for age and gender with controls at a 1:4 ratio and 108,149 patients without PD were enrolled in the control cohort. In total, 135,190 individuals were enrolled in our study and followed to investigate the incidence of SS. Table 1 lists the demographic data on both cohorts; there were no significant differences in age and gender. Most participants were in a low income bracket and resided in highly urbanized areas and in northern Taiwan. Patients with PD had higher rates of comorbidities other than alcoholism, including DM, hypertension, hyperlipidemia, coronary arterial disease, stroke, obesity and tobacco use disorder.

The average follow-up duration was 6.91 ± 0.65 years for the PD cohort and 6.94 ± 0.57 years for the control cohort. Finally, 3292 (2.44%) patients had newly diagnosed SS and patients with PD had a higher incidence of SS (3.34% vs. 2.21%, crude HR = 1.52, 95% CI: 1.41–1.64, Table 2). After adjustment for confounding factors, Cox proportional hazards regression was performed to evaluate the independent risk of subsequent SS and the results are shown in Table 3. The aHR for PD was 1.47 (95% CI: 1.36–1.59, *p* < 0.05, Table 3). Additionally, males had a significantly lower risk of SS (aHR: 0.36% CI: 0.33–0.39, *p* < 0.05). Individuals with high income and those who resided in central Taiwan were at higher risk of SS. Patients with hypertension, hyperlipidemia, stroke, alcoholism and smoking were at lower risk of subsequent SS.

## 4. Discussion

Our study is the first to investigate the relationship between PD and subsequent SS. Xerostomia is common in both diseases and immune-mediated inflammatory processes are involved in both diseases. We found an approximately 50% increased risk of newly diagnosed SS in patients with PD. Physicians should be aware of symptoms and signs of SS in patients with PD, such as dry mouth and dry eyes.

Xerostomia is a cardinal symptom of SS and is common in patients with PD. Several studies have investigated the incidences of PD in patients with SS. Patients with SS had a significantly higher risk of PD [24,25,31,32,33]. The observed risk may be bidirectional, and our study provided a scientific evidence supporting increased risk of SS in patients with PD. Several factors may contribute to the observed increase in risk. First, oral hygiene and overgrowth of microorganisms may contribute to the association. In patients with SS, destruction of the salivary glands results in dry mouth and bacterial overgrowth and is followed by PD. Periodontal treatment in patients with SS may increase salivary flow and improve clinical and immunological parameters and quality of life [34]. Furthermore, alterations of cytokine network may play important roles in the link between PD and SS. Alterations of the cytokine profile in serum or saliva have been reported in patients with PD, including IL-1, IL-6, IL-10, IL-17A, IL-17F, IL-22, IL-25, IL-33, tumor necrosis factor-alpha and interferon-gamma [17,18,35,36,37]. An increased risk of systemic diseases, such as cardiovascular diseases, diabetes, rheumatoid arthritis, preeclampsia, preterm birth and ulcerative colitis, has been reported in previous studies [14,20,21,22,23]. Similarly, obvious dysregulation of the cytokine network has been observed in patients with SS and cytokines may be considered as potential therapeutic targets [2,6,38,39]. In patients with SS, overexpression of pro-inflammatory cytokines, including interferon-γ, IL-12, IL-18, IL-6 and B-cell activating factor, was found. In contrast, anti-inflammatory cytokines, such as IL-4 and transforming growth factor-β, were downregulated. However, the complete mechanisms underpinning PD and SS are complicated and not fully understood and we found an obvious increased risk of SS in patients with PD. An association may exist between these two diseases and further studies are warranted to clarify the underlying pathophysiology.

The cardinal symptoms of SS are dry mouth and dry eyes and are easily overlooked. Delayed diagnosis of SS is not uncommon [2]. The Sjögren’s Syndrome Foundation launched a 5-year Breakthrough Goal in January 2012 and its aim was to reduce the duration between the onset and diagnosis of Sjögren’s syndrome by 50% in 5 years [40]. They showed that the average period from symptom onset to diagnosis was approximately 6 years. The average follow-up duration in our study was 6.91 ± 0.65 years for the PD cohort, compatible with previous reports. Dry mouth in patients with PD may be an early presentation of SS. Reducing the time from onset to diagnosis is valuable and our study found a statistically increased risk of SS in patients with PD. Physicians should educate their PD patients and be aware of the symptoms of SS. If PD patients have one of the following symptoms: dry eyes, skin rash, joint pain, salivary gland swelling or any other symptom of SS, further investigation for SS is recommended.

After adjustment for confounding factors, our study found a decreased risk of subsequent SS in patients with hypertension, hyperlipidemia, stroke, alcoholism and smoking (Table 3). The association between SS and other systemic disease is complicated. Chiang et al. investigated the risk of stroke in patients with SS and no obvious association was found. The relationship between smoking and SS was reported previously and anti-inflammatory effects of cigarettes may contribute to the observed reduction of risk [41,42]. However, the adverse effects of smoking are undoubtedly versatile and huge and smoking is not encouraged. Again, the underlying biological mechanisms of SS and related systemic diseases are complex and further studies are required.

As the NHIRD is a nationwide database with broad coverage, the findings of our study are representative of the general population and clinically significant. However, our study is subject to some limitations. First, laboratory test results are not available in our database. Although SS is a catastrophic illness in Taiwan and the diagnosis is accurate and verified, it is valuable to explore inflammatory markers and disease subgroups and to investigate the possible mechanism behind the observed association. Second, patients with different severities of PD may have different risk levels of subsequent SS. Details regarding PD severity would contribute to further clarification of the increased risk. However, classification of PD severity relied on the detailed local findings, but related information was not complete in present database. Therefore, the severity of SS and treatment responses were not analyzed. Although a 13-year total study period is not short, long-term prognosis and treatment may be different. Similarly, edentulous disease (teeth loss) is common in patients with severe PD and overlaps the pathophysiology of PD [43]. Complete information of teeth loss in each individual was not available and we did not exclude patients with edentulous disease. It is valuable to compare the risks of SS in patients with different severity of PD and different edentulous conditions. Moreover, the entire mechanism of SS is not completely understood and further studies are required to elucidate the underlying pathophysiology linking PD and SS.

## 5. Conclusions

In conclusion, this large-scale, nationwide, population-based study found that patients with PD have an approximately 50% increased risk of subsequent SS. An immune-mediated inflammatory response may contribute to the association. Physicians should be aware of the symptoms and signs of SS in patients with PD and appropriate investigations of SS may contribute to an early diagnosis of SS.

## Figures and Tables

**Table 1:** Distribution of gender, age groups and comorbidities in individuals with and without periodontal disease (PD).

Variables		Number of Individuals		
		PD Cohort	Control Cohort	*p* Value
		*N* = 27,041	*N* = 108,149	
Gender				
	Female	13,068 (48.3%)	52,267 (48.3%)	0.995
	Male	13,973 (51.7%)	55,882 (51.7%)	
Age Groups				1
	20–29	4524 (16.7%)	18,096 (16.7%)	
	30–39	4567 (16.9%)	18,268 (16.9%)	
	40–49	6557 (24.2%)	26,228 (24.3%)	
	50–59	5746 (21.2%)	22,972 (21.2%)	
	60–69	3131 (11.6%)	12,519 (11.6%)	
	≥70	2516 (9.3%)	10,066 (9.3%)	
Income Groups				<0.001
	<20,000	17,429 (64.5%)	78,921 (73%)	
	20,000–39,999	4964 (18.4%)	17,995 (16.6%)	
	40,000–59,999	3032 (11.2%)	8090 (7.5%)	
	≥60,000	1616 (6%)	3143 (2.9%)	
Geography				<0.001
	North	13,351 (49.4%)	55,469 (51.3%)	
	Central	5467 (20.2%)	18,679 (17.3%)	
	South	7570 (28%)	31,141 (28.8%)	
	Other	653 (2.4%)	2860 (2.6%)	
Urbanization level				<0.001
	1 (highest)	14,199 (52.5%)	47,711 (44.1%)	
	2	6613 (24.5%)	28,190 (26.1%)	
	3	4543 (16.8%)	22,174 (20.5%)	
	4 (lowest)	1686 (6.2%)	10,074 (9.3%)	
Comorbid diseases				
Alcoholism		494 (1.8%)	2268 (2.1%)	<0.05
CAD		5419 (20%)	15,865 (14.7%)	<0.001
	DM	6214 (23%)	18,835 (17.4%)	<0.001
	Hyperlipidemia	9711 (35.9%)	28,319 (26.2%)	<0.001
	Hypertension	10,305 (38.1%)	34,531 (31.9%)	<0.001
	Obesity	439 (1.6%)	1318 (1.2%)	<0.001
	Smoking	4368 (16.2%)	12,237 (11.3%)	<0.001
	Stroke	3447 (12.7%)	10,921 (10.1%)	<0.001

Note: Abbreviations: CAD: coronary artery disease; DM: diabetes mellitus; PD: periodontal disease.

**Table 2:** Association between PD and Sjögren’s syndrome (SS) analyzed by employing a Cox regression model.

Disease Incidence	Number of Individuals	
	PD Cohort	Control Cohort
	*N* = 27,041	*N* = 108,149
With Sjogren’s syndrome	903 (3.34%)	2389 (2.21%)
Without Sjogren’s syndrome	26,138 (96.66%)	105,760 (97.79%)
Crude hazard ratio	1.52 (1.41 to 1.64) ‡	

‡ *p* < 0.001 for comparison between patients with two groups.

**Table 3:** Independent predictors of SS identified by Cox regression analysis.

Variables		Crude	Adjusted
		HR (95% CI)	HR * (95% CI)
PD		1.52 (1.41 to 1.64) ‡	1.47 (1.36 to 1.59) ‡
Gender			
	Female	1	1
	Male	0.37 (0.34 to 0.4) ‡	0.36 (0.33 to 0.39) ‡
Age Groups			
	20–29	1	1
	30–39	0.86 (0.75 to 0.98) †	0.91 (0.8 to 1.04)
	40–49	0.99 (0.88 to 1.11)	1.17 (1.04 to 1.32) †
	50–59	1.39 (1.24 to 1.55) ‡	1.75 (1.55 to 1.97) ‡
	60–69	1.62 (1.43 to 1.83) ‡	2.28 (1.99 to 2.61) ‡
	≥70	1.01 (0.88 to 1.18)	1.64 (1.4 to 1.92) ‡
Income Groups			
	<20,000	1	1
	20,000–39,999	1.16 (1.06 to 1.26) †	1.26 (1.14 to 1.38) ‡
	40,000–59,999	1.04 (0.91 to 1.18)	1.34 (1.17 to 1.52) ‡
	≥60,000	1.2 (1.01 to 1.43) †	1.58 (1.32 to 1.89) ‡
Geography			
	North	1	
	Central	2.08 (1.93 to 2.25) ‡	2.24 (2.06 to 2.44) ‡
	South	0.78 (0.72 to 0.86) ‡	0.82 (0.75 to 0.9) ‡
	Other	0.73 (0.56 to 0.95) †	0.81 (0.62 to 1.07)
Urbanization level			
	1 (highest)	1	1
	2	0.91 (0.83 to 0.99) †	0.84 (0.77 to 0.91) ‡
	3	0.93 (0.85 to 1.02)	0.84 (0.77 to 0.93) †
	4	0.89 (0.78 to 1.01)	0.88 (0.77 to 1.01)
Comorbid diseases			
Alcoholism		0.3 (0.19 to 0.45) ‡	0.48 (0.31 to 0.74) †
CAD		1.08 (0.99 to 1.18)	1.07 (0.97 to 1.19)
DM		0.95 (0.87 to 1.03)	0.92 (0.83 to 1.02)
Hyperlipidemia		0.96 (0.89 to 1.04)	0.86 (0.79 to 0.95) †
Hypertension		0.96 (0.9 to 1.04)	0.86 (0.78 to 0.94) †
Obesity		0.87 (0.63 to 1.2)	0.86 (0.63 to 1.19)
Smoking		0.73 (0.65 to 0.82) ‡	0.82 (0.73 to 0.92) †
Stroke		0.86 (0.76 to 0.96) †	0.82 (0.73 to 0.93) †

* Each variable was adjusted for every other variable. Abbreviations: CAD: coronary artery disease; DM: diabetes mellitus; HR: hazard ratio; PD: periodontal disease. † *p* < 0.05 for comparison between patients with two groups. ‡ *p* < 0.001 for comparison between patients with two groups.
